# Functional Analysis of Genetic Variations in Surfactant Protein D in Mycobacterial Infection and Their Association With Tuberculosis

**DOI:** 10.3389/fimmu.2018.01543

**Published:** 2018-07-02

**Authors:** Miao-Hsi Hsieh, Chih-Ying Ou, Wen-Yu Hsieh, Hui-Fang Kao, Shih-Wei Lee, Jiu-Yao Wang, Lawrence S. H. Wu

**Affiliations:** ^1^Institute of Basic Medical Science, College of Medicine, National Cheng Kung University, Tainan, Taiwan; ^2^Institute of Clinical Medicine, College of Medicine, National Cheng Kung University, Tainan, Taiwan; ^3^Institute of Biochemistry and Molecular Biology, College of Medicine, National Cheng Kung University, Tainan, Taiwan; ^4^Allergy and Clinical Immunology Research (ACIR) Center, College of Medicine, National Cheng Kung University, Tainan, Taiwan; ^5^Chest Medicine, General Taoyuan Hospital, Taoyuan, Taiwan; ^6^Institute of Respiratory Research, The First Affiliated Hospital of Guangzhou Medical University, Guangzhou, China; ^7^Graduate Institute of Biomedical Sciences, China Medical University, Taichung, Taiwan

**Keywords:** infection, *Mycobacterium*, single-nucleotide polymorphisms, surfactant protein D, tuberculosis

## Abstract

Surfactant proteins (SPs)-A and -D are C-type lectins of the collectin family and function in the clearance of infectious particles in the lungs. Some polymorphisms of SPs that give rise to amino acid changes have been found to affect their function. Several SP-A gene polymorphisms have been reported to be associated with respiratory infection diseases, such as tuberculosis (TB). However, the relationship between surfactant proteins D (SP-D) polymorphisms and TB is still unclear. To study the associations between SP-D polymorphisms and TB, the correlations of SP-D polymorphisms with TB were examined in a case–control study, which included 364 patients with TB and 177 control subjects. In addition, we cloned two major SP-D exonic polymorphism C92T (rs721917) and A538G (rs2243639) constructs and used these for *in vitro* assays. The effects of SP-D polymorphisms on agglutination and other interactions with *Mycobacterium bovis* bacillus Calmette–Guérin (*M. bovis* BCG) were evaluated. In comparison with SP-D 92C (amino acid residue 16, Threonine), our results showed that SP-D 92T (amino acid residue 16, Methionine) had a lower binding ability to *M. bovis* BCG, a lower capacity to inhibit phagocytosis, lesser aggregation, poorer survival of bacillus Calmette–Guérin (BCG)-infected MH-S cells, and less inhibition of intracellular growth of *M. bovis* BCG. The case–control association study showed that the 92T homozygous genotype was a risk factor for TB. However, a lesser effect was seen for polymorphism A538G. In conclusion, the results of functional and genetic analyses of SP-D variants consistently showed that the SP-D 92T variant increased susceptibility to TB, which further confirmed the role of SP-D in pulmonary innate immunity against mycobacterial infection.

## Introduction

Pulmonary tuberculosis (TB), caused by *Mycobacterium tuberculosis* (*MTB*), is a global public health issue, as it is the leading cause of death among infectious diseases in many countries. Though TB has diminished over a long period of time, it has reappeared and has become a serious concern once again in many regions. The resurgence of TB is also accompanied by increasing numbers of cases of multidrug-resistant TB due to the development of extensively drug-resistant bacterial strains (1, 2). A study estimated that more than 30% of the world’s population has been infected with *MTB*, and the infection kills approximately two million people annually (3). In Taiwan, there were more than 12,000 cases (55/100,000 population) of TB in 2011 alone, and at least 600 deaths (2.8/100,000 population) were caused by TB (4). The development of and susceptibility to TB involves complicated host environment–pathogen interactions, and genetic components have been suggested to be involved in the process. Recently, more evidence has been uncovered to indicate that individuals with certain genetic chrematistics are particularly vulnerable to TB. In addition, data accumulated from different types of studies, such as twin studies (5, 6), genome-wide linkage analyses (7–10), and genome-wide association studies (11–13), have demonstrated that individuals with particular genetic variations are at high risk of TB (14). In fact, among people infected with TB, only 10% develop active pulmonary TB, implying that polymorphisms of genes associated with host immune responses may be key to the development of pulmonary TB (15).

C-type lectins, collagen-like calcium-dependent pulmonary collectins, are some of the molecules correlated with innate host immune responses. These include lung surfactant proteins (SP)-A and -D and pattern recognition molecule–mannose-binding lectin/protein (MBL or MBP) (16). These molecules play important roles in the host cellular response against mycobacterial infections (17). During the course of *MTB* infection, SP-A mediates the adherence of *MTB* to human alveolar macrophages and induces mannose receptor activity, which further increases their phagocytic uptake of *MTB* (18). Surfactant proteins D (SP-D), however, directly interacts with *MTB*, resulting in reduction of *MTB* phagocytosis (19), and the inhibition effect on *MTB* uptake by macrophages is not associated with the bacterial agglutination process (20). Incubation of *MTB* with SP-D results in reduced uptake of the bacteria by macrophages (19) and limits *MTB* growth inside cells by facilitating the fusion of *MTB* phagosomes with lysosomes of cells (21). Allelic variations of SP-A and SP-D genes have been reported to be target candidates in several infectious diseases, including TB, as they are directly involved in the process of lung pathogen clearance (22). A study of patients of a Mexican population showed that several SP flanking marker alleles are associated with an increased risk of susceptibility to TB (23). Intronic single-nucleotide polymorphisms (SNPs) in the collagen region of SP-A2 and intragenic SNPs in SP-A1 and SP-A2 have also been demonstrated to contribute to the risk of susceptibility to TB (24, 25). Thus, based on the aforementioned research and other previous studies, it has been proposed that surfactant polymorphisms may alter splicing regulation and impact on mRNA maturation (25). However, many of the underlying mechanisms are still unclear. Recently, Yang et al. (26) reported that SNPs in SP-A, particularly at alleles of amino acids 91 (G) and 140 (T), are associated with TB in the Han population in China. Studies of protein constitution in bronchoalveolar lavage and surfactant gene polymorphisms have suggested that SP-D is directly linked to several pulmonary inflammatory diseases (16, 27). SP-D polymorphisms have also been found to be connected to severe respiratory syncytial virus infection, with the rs721917 SP-D allele coding for methionine (28). Our previous study also showed that genetic variants of pulmonary SP-D could determine serum levels of SP-D and predict the outcome of chronic obstructive pulmonary disease in a Chinese population (29). To the best of our knowledge, only one study of a Mexican population has identified an association between SP-D polymorphisms and susceptibility to TB (23). However, this research only investigated selective loci, without whole SP-D gene analysis, and lacked functional study of the correlations between SP-D polymorphisms and *MTB* infection. In fact, little comprehensive work has been carried out to analyze the relationships between genetic variants of SP-D proteins and host immunity against *MTB* infection.

In this study, the effects of functional (in exon, non-synonymous) polymorphisms of SP-D on the interaction between SP-D and *Mycobacterium bovis* bacillus Calmette–Guérin (*M. bovis* BCG) were investigated. Furthermore, the associations between SP-D functional polymorphisms and susceptibility to TB were also investigated. This work provides evidence that utilizing functional DNA variants of SP-D in clinical association studies, instead of markers of SNPs, advances our understanding of surfactant genetic susceptibility and innate defense mechanisms to pulmonary TB and other lung diseases.

## Materials and Methods

### Clinical Sample Collection

A total of 364 patients who were treated for active TB at the General Taoyuan Hospital (Taoyuan, Taiwan) and National Cheng Kung University Hospital (Tainan, Taiwan) between 2010 and 2012 were surveyed consecutively. The inclusion criteria were: adult patients newly diagnosed with active TB, evident lesions of TB on simple X-ray and computed tomography images, and positive results of sputum smears and cultures for mycobacteria. Patients with HIV infection were excluded from this study. The control group comprised 177 volunteer subjects without active TB or a history of TB. Written informed consent was obtained from each patient and volunteer enrolled in this study. The study protocol conformed to the ethical guidelines of the 1975 Declaration of Helsinki and was approved by the Ethics Committees of Taoyuan General Hospital and National Cheng Kung University Hospital.

### DNA Extraction and SNP Genotyping

Genomic DNA was extracted from oral swabs collected from the enrolled TB patients and non-TB subjects using a QIAamp DNA Mini Kit (QIAGEN, Valencia, CA, USA) according to the manufacturer’s instructions. The extracted genomic DNA was analyzed using agarose gel electrophoresis, quantitatively determined by spectrophotometry, and stored at −80°C until use. Genotyping was performed on the two major non-synonymous SNPs of SP-D, rs721917 (C92T) and rs2243639 (A538G), for genetic association analysis. The SNPs were genotyped using the high-throughput, 384-microtiter plate MassARRAY System, SEQUENOM, according to the manufacturer’s protocol. In brief, DNA containing the SNP site of interest was amplified, followed by performance of the homogenous MassEXTEND assay, in which label-free primer extension chemistry was used to generate allele-specific diagnostic products. The allele-specific diagnostic products have a unique molecular weight, and this can be distinguished through the application of matrix-assisted laser desorption ionization time-of-flight mass spectrometry.

### Chemicals and Plasmids

QIAprep Miniprep and QIAGEN plasmid Midi Kit for plasmid preparation were obtained from QIAGEN. Restriction enzymes including *Hind* III and *Sac* II were obtained from TaKaRa Bio. *Dpn* I for site mutagenesis was obtained from New England Biolabs. The vector pcDNA3.1/myc-His B was purchased from Invitrogen.

### Site-Directed Mutagenesis of Human SP-D

The cDNA fragment encoding human SP-D was amplified by PCR using primers (forward: 5′-CCCAAGCTTGCCATGCTGCTCTTCCTCCTCTCTG-3′ containing a 5′ HindIII adaptor; reverse: 5′-GCTCTAGATCAGAACTCGCAGACCACAAG-3′ containing a 5′ SacII adaptor) and cloned into HindIII and SacII sites, leading to the formation of recombinant plasmid pcDNA3.1/myc-His B-SP-D (92T/538G). Site-directed mutagenesis was performed using mutagenic primers of SP-D 92T (forward: 5′-GACCTACTCCCACAGAACAACGCCCAGTGCTTG-3′; reverse: 5′-CAAGCACTGGGCGTTGTTCTGTGGGAGTAGGTC-3′ and SP-D538G forward: 5′-GTGGAGTCCCTGGAAACACAGGGGCAGC-3′; reverse: 5′-CAAGCACTGGGCGTTGTTCTGTGGGAGTAGGTC-3′). The recombinant plasmids were then transformed into *Escherichia coli* XL-1 Blue competent cells (Invitrogen) by the heat shock method, and grown in LB broth at 37°C. Colonies of transformed cells were selected for further gel electrophoresis.

### Construction and Expression of Recombinant SP-D (rSP-D) Site-Directed Mutants

**Figure 1:**
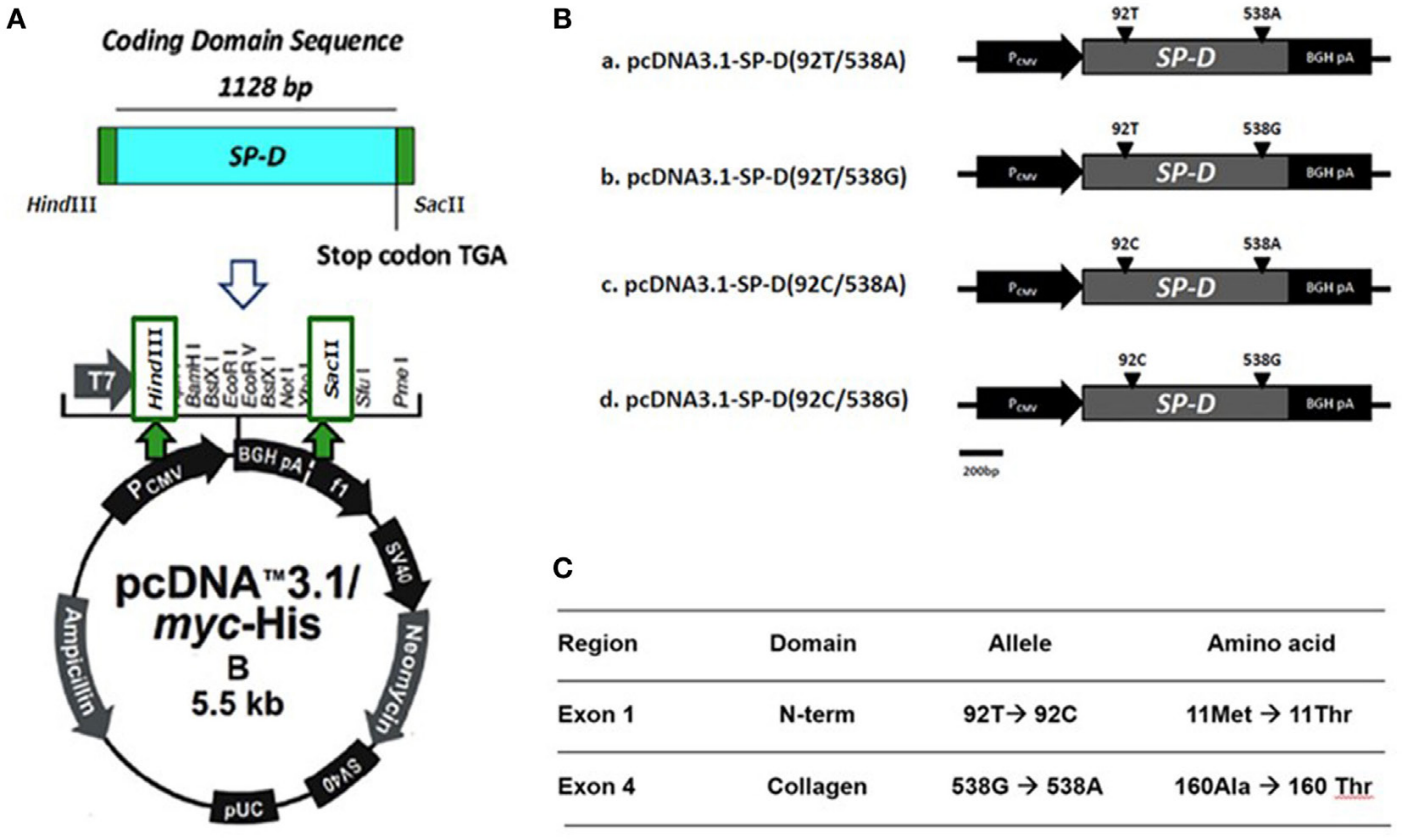
**(A)** Illustration of the construction of recombinant full-length human surfactant proteins D (SP-D) plasmid into which the SP-D gene was inserted at the multiple cloning site of a pcDNA 3.1/myc-His B vector between *Hind* III and *Sac* II. **(B)** Four recombinant SP-D (rSP-D) plasmids that carried two major single-nucleotide polymorphisms under four different combinations (92T/538A, 92T/538G, 92C/538A, and 92C/538G) were created using gene cloning and site-directed mutagenesis. **(C)** Corresponding sites of nucleotides and amino acids in different polymorphisms of SP-D variants.

**Figure 2:**
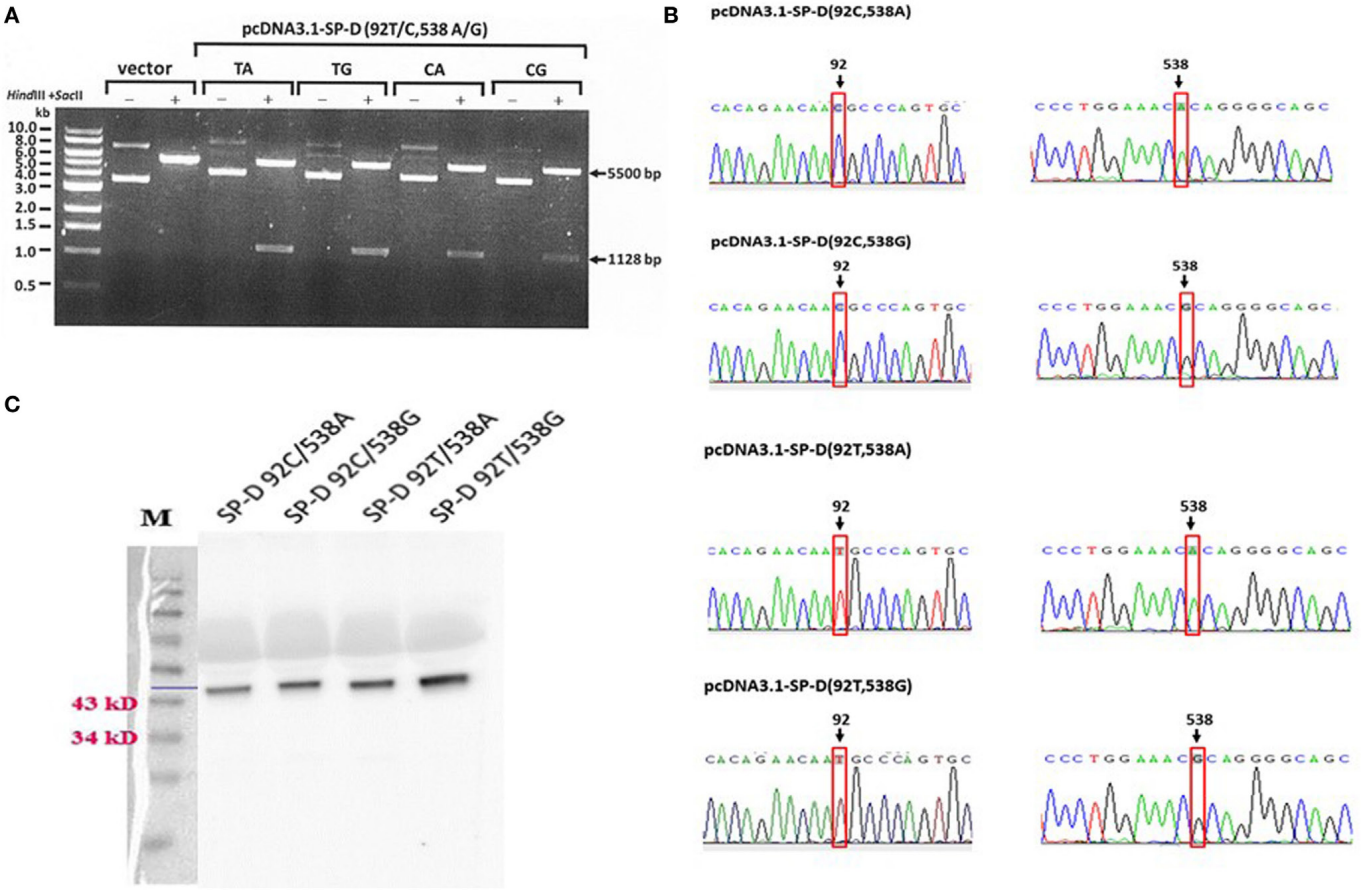
**(A)** Double digestion of the vector and four surfactant proteins D (SP-D) gene variant plasmids using the restriction enzymes *Hind* III and *Sac* II for diagnostic digestion (TA: 92T/538A, TG:92T/538G, CA: 92C/538A, and CG: 92C/538G). **(B)** Sequencing chromatograms of the pcDNA3.1-SPD variant constructs confirmed the generation of four recombinant SP-D (rSP-D) genes using site-directed mutagenesis. **(C)** Western blots of the four rSP-D proteins from transfected HEK 293 cells showing the correct sizes of the proteins.

The construction of recombinant plasmid pcDNA3.1/myc-His B-SP-D (92T/538G) is illustrated in Figure 1A. Site-directed mutagenesis of pcDNA3.1/myc-His B-SP-D (92T/538G) resulted in four variants (Figure 1B). SNPs in residues 92 and 538 resulted in changes in amino acids (Figure 1C). Gel electrophoresis of the four mutagenic DNA products after transformation with *Hind* II and *Sac* II diagnosis digestion is demonstrated in Figure 2A. The results of DNA sequencing of the four variants of SP-D are illustrated in Figure 2B. Expression and quantification of the four rSP-D site-directed mutants were confirmed by western blot (Figure 2C) and SP-D ELISA analysis (data not shown).

### Expression and Quantification of Four Variants of rSP-D

Four variants of the rSP-D gene were transfected into human embryonic kidney 293 (HEK 293) cells (ATCC No. CRL-1573) using Lipofectamine LYX™ Reagent (Invitrogen). Culture medium (serum-free Dulbecco’s Modified Eagle Medium) from transfected HEK 293 cells was collected for further western blot analysis. An SP-D ELISA kit (Hycult) was used for final quantification of the four rSP-D variants.

### Cell and Bacterial Culture

The alveolar macrophage cell line derived from BALB/c mice (MH-S cells; American Type Culture Collection, Rockville, MD, USA) used in the study was a continuous cell line of murine alveolar macrophages established after transformation of cells obtained by bronchoalveolar lavage from BALB/c mice with simian virus 40. The cells were cultured in RPMI 1640 medium (Gibco BRL, Gaithersburg, MD, USA) supplemented with 10% fetal bovine serum, 50 U/ml penicillin, 0.05 mg/ml streptomycin, 20 mM l-glutamine, 10 mM HEPES, 10 mM sodium pyruvate, 1% glucose, and 50 µM 2-mercaptoethanol at 37°C in 5% CO_2_/95% air. *M. bovis* BCG was cultured on Middlebrook 7H11 agar (BD) at 37°C in an incubator. Bacteria were harvested, and pellets were suspended in PBS plus 10% glycerol. Aliquots (~5 × 10^8^/vial) were stored at −80°C. Before infection, the bacteria supplemented with glycerol were replaced with a final concentration of 0.05% v/v Tween 80 and 2 mM CaCl_2_ PBS to reduce bacillus Calmette–Guérin (BCG) clumping and for subsequent preparation for SP-D binding. To avoid effects on the viability of *M. bovis* BCG, penicillin and streptomycin were not added to the culture medium of the MH-S cells (Sigma-Aldrich, St. Louis, MO, USA) that were prepared for infection.

### *M. bovis* BCG Infection

A multiplicity of infection (MOI) of 1 (1 bacterium per MH-S cell) was used for all the experiments, with the exception of the phagocytosis assay, in which an MOI of 5 was used. Quantitative bacteria in different cell culture plates (6-well: 10^5^, 12-well: 5 × 10^4^, 24-well: 2.5 × 10^4^, and 96-well: 5 × 10^3^) were supplemented with 100 mM glucose or 100 mM maltose, with or without 10 mM EDTA, and with one of the four variants of SP-D (0.5 or 1 µg/ml). One hour after infection, un-infected MH-S cells were washed twice with PBS, and the same conditional medium was used for subsequent analysis.

### Solid-Phase Bacterial ELISA

Lipoarabinomannan (LAM) from *M. bovis* BCG, which belongs to the class of LAMs capped with mannosyl residues, has a similar structure and immune-regulatory function to *MTB* (19). LAM also serves as a major binding molecule for SP-D (30). Based on this, we examined the roles of the four variants of rSP-D in the binding to *M. bovis* BCG. In experiments designed to determine the saturable binding activity of the four SP-D variants to *M. bovis* BCG, bacterial suspensions (10^6^ bacteria in 100 µl TBS) were first dried at 37°C in an incubator for 7 h, and then exposed to ultraviolet light for 2 h to kill any viable bacteria. After blocking with 3% BSA overnight at 4°C, 1 and 10 µg/ml of the four variants of SP-D in PBS with 2 mM CaCl_2_ were added with or without 10 mM EDTA, and with 100 mM maltose or 100 mM glucose. The results from 1 and 10 µg/ml of the four variants of SP-D were similar, then 1 µg/ml was used to further experiments. After incubation for 1 h at room temperature, the bacterial suspensions were washed and then incubated with a 1:5,000 dilution of anti-SP-D antibody for 2 h at room temperature. After incubation, the suspensions were washed again and then incubated with a 1:3,000 dilution of goat anti-mouse IgG conjugated to HRP for 2 h at room temperature. Finally, TMB substrate was added after washing. Substrate development was stopped after 12 min by 2N H_2_SO_4_, and the absorbance of individual wells was determined at O.D. 450 nm using a SpectraMax M2 ELISA reader (Molecular Devices, Sunnyvale, CA, USA).

### Phagocytosis of *M. bovis* BCG by MH-S Cells

A Vybrant Phagocytosis Assay Kit (V6694; Invitrogen™, Thermal Fisher Scientific) was used to measure the phagocytosis of mycobacteria by MH-S cells. Briefly, bacteria (10^9^/ml) were labeled by incubation with FITC (Sigma-Aldrich) at 1 µg/ml in PBS for 2 h at 37°C. Thereafter, FITC-labeled bacteria were washed twice with PBS, then opsonized, and used for infection as described above. Macrophages infected by FITC-labeled *M. bovis* BCG were analyzed and gated using flow cytometry to assess the effects of the four rSP-D variants on the phagocytosis of MH-S cells. Briefly, MH-S cells (10^5^) were incubated with FITC-labeled *M. bovis* BCG (5 × 10^5^ CFU) in a 6-well cell culture plate (BD, Franklin Lakes, NJ, USA) in the absence or presence of the four SP-D variants at concentrations ranging from 0.5 to 1 µg/ml. After incubation for 1 h, MH-S cells were scraped out after fixation with 4% formaldehyde PBS (pH 7.2) and examined using a flow cytometer (FACSCalibur; BD, San Jose, CA, USA) equipped with an argon laser that emits at 488 nm. At least 1 × 10^4^ of cells with FITC-labeled bacteria were counted in the FACS analysis.

### Agglutination and Cell Migration of *M. bovis* BCG-Infected MH-S Cells

To further characterize the effects of the four SP-D variants on the agglutination of MH-S cells after mycobacterial infection as described above, micrographs were taken 2 days after infection using an Olympus IX-71 inverted microscope. The area of the agglutinates was measured using ImageJ 1.39g software (National Institutes of Health, Bethesda, MD, USA). The diameter was estimated from the area based on the circular form of the aggregates according to the formula Area = (*R* × *D*^2^)/4, where *R* = ratio of a circle’s circumference to its diameter and *D* = diameter. To determine the effects of the four variants of SP-D on macrophage cell migration, 2.5 × 10^4^ MH-S cells were seeded in a 24-well cell culture plate and infected with *M. bovis* BCG as described above. The culture medium was harvested 2 days after infection and added into a new 24-well cell culture plate equipped with Millicell Hanging Cell Culture Inserts (3 µm pore size, Millipore). Each insert was seeded with 5 × 10^4^ MH-S cells. After 1 day of culture, the inserts were removed and observed using an Olympus IX-71 inverted microscope to count the cells migrated through the membrane. The chemoattractive MH-S cells were quantified using ImageJ 1.39g software.

### *M. bovis* BCG-Infected MH-S Cells Survival Assay

Quantitative bacteria in a 96-well cell culture plate (5 × 10^3^ MH-S/100 μl medium) were infected with *M. bovis* BCG as described above. Two days after infection, surviving MH-S cells were evaluated using a Cell Counting Kit-8 (CCK-8) (Dojindo Laboratories, Kumamoto, Japan) with a SpectraMax M2 ELISA reader (Molecular Devices).

### *M. bovis* BCG Intracellular Growth Assay

To determine the effects of the four SP-D variants on the intracellular viability of bacteria after phagocytosis by macrophages, 100 µl RIPA buffer (10×, Millipore) were added to the culture plate 2 days after MH-S cells had been infected (5 × 10^4^ in a 12-well cell culture plate). Aliquots of MH-S cell lysate were then plated onto 7H11 agar (BD) to quantify the intracellular growth of viable *M. bovis* BCG. Colony-forming units were counted after 28 days of bacteria culture.

### Statistical Analysis

Results are given as means ± SEs. ANOVA was used to compare the rSP-Ds. All *P* values less than 0.05 were considered significant. Statistical analysis was carried out using Prism, version 5 (GraphPad Software, San Diego, CA, USA). In the genetic association study, the quality of the genotype data was evaluated by Hardy–Weinberg equilibrium proportion tests. χ^2^ tests were used for association analyses. Odds ratios and 95% confidence intervals were calculated from contingency tables. SNPs showing significant associations (*P* value ≤ 0.05) in the tests were further evaluated using logistic regressions adjusted for age and gender in odds ratio analysis. Statistical analyses for the association study were performed using SPSS 17.0 software (SPSS Inc., Chicago, IL, USA).

## Results

### Subject Characterization

Surfactant proteins D genetic polymorphisms are most often associated with pulmonary diseases, and our hypostatized genetic variants of SP-D may have different levels of contribution to the pathogenesis of TB. Therefore, we analyzed the genotype and allele frequencies of SP-D in TB- and non-TB-infected patients. 364 case subjects with diagnosed TB and 177 control subjects without a history of TB infection were enrolled. The mean age of the cases and controls was 55.22 and 57.75 years, respectively (Table 1). Cases were more likely to be male (*P* < 0.0001). There was no significant difference between the groups regarding age (*P* > 0.05).

**Table 1:** Demographic data of the study subjects.

	Tuberculosis (TB)	Non-TB	
Age (years, mean ± SD)	55.22 ± 20.09	57.75 ± 11.08	*P* = 0.060 (*t* = 1.884)
**Gender**			
Male	256	108	*P* < 0.0001 (χ^2^ = 30.600)
Female	81	96	

### Variants of SP-D Associated With TB

The genotype and allele frequencies of C92T (SNP ID: rs721917) and A538G (SNP ID: rs2243639) could not be derived from the Hardy–Weinberg principle. The association between TB and rs721917 genotypes was marginally significant (*P* = 0.050), and this significance was reduced after adjusting for age and gender (Table 2). However, odds ratio analysis showed that subjects with TT homozygous rs721917 alleles had an increased risk of susceptibility to TB (OR = 1.95, *P* = 0.045; Table 2), as the homozygous methionine amino acid at SP-D polypeptide position 11 was more frequent in TB patients than in non-TB subjects. The statistically significant association between rs2243639 and TB was increased when the results were adjusted for age and gender (from 0.061 to 0.033; Table 2). The homozygous AA (amino acid 160 threonine) genotype of rs2243639 was protective against TB.

**Table 2:** Genotyping results and odds ratio analysis of the associations between surfactant proteins D single-nucleotide polymorphisms (SNPs) and tuberculosis (TB).

SNP/genotype	TB	Non-TB	*P*	Adj.*P*	Adj. OR (95% confidence interval)	*P*
**rs721917 (C92T)**						
CC (ref.)	134 (36.8%)	80 (45.2%)	0.050	0.104		
CT	176 (48.6%)	82 (46.3%)	χ^2^ = 5.996	χ^2^ = 4.519	1.27 (0.85, 1.86)	0.240
TT	54 (14.8%)	15 (9%)			1.95 (1.01, 3.75)	0.045
**rs2243639 (A538G)**						
GG (ref.)	263 (72.2%)	129 (72.9%)	0.061	0.033		
AG	96 (26.4)	40 (22.6%)	χ^2^ = 5.587	χ^2^ = 6.853	1.15 (0.74, 1.78)	0.545
AA	5 (1.4%)	8 (1.4%)			0.23 (0.07, 0.75)	0.015

*Adj., adjusted by age and gender using logistic regression; ref., reference genotype*.

### Binding Activities of Four Variants of rSP-D to *M. bovis* BCG

**Figure 3:**
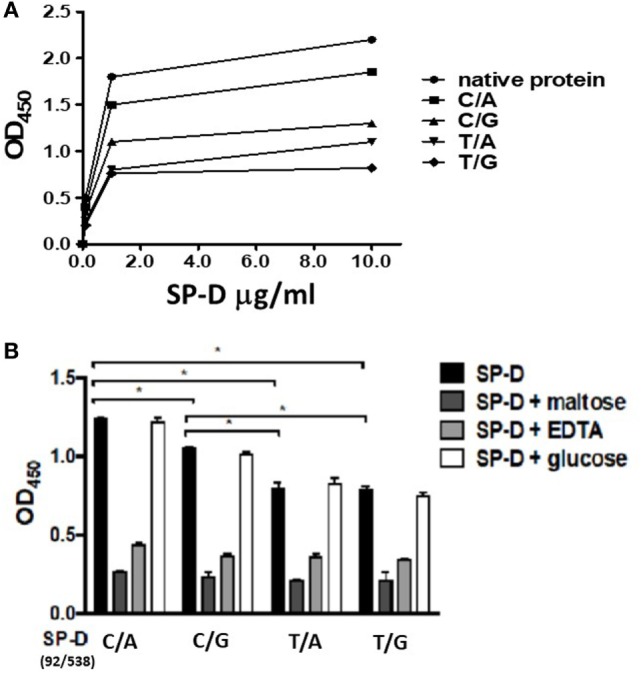
**(A)** Dose-binding curves of native and four genetic variants of recombinant SP-D (rSP-D) proteins to *Mycobacterium bovis* bacillus Calmette–Guérin (*M. bovis* BCG). rSP-D protein (1 and 10 µg/ml) were added to wells coated with ultraviolet-killed *M. bovis* BCG (10^6^ bacteria), then quantitated using solid-phase bacterial ELISA. **(B)** Inhibition effects of maltose and EDTA on the binding activity of four variants of rSP-D to *M. bovis* BCG. Surfactant proteins D (SP-D) protein (1 µg/ml) in PBS with 2 mM CaCl_2_ was added into *M. bovis* BCG-coated wells alone, or with 10 mM EDTA, or 100 mM maltose, or 100 mM glucose. The binding activity to *M. bovis* BCG of rSP-D with residue 11Thr [(92C/538A) and (92C/538G)] was significantly higher than that of rSP-D with residue 11Met [(92T/538A) and (92T/538G)] (*P < 0.05). C/A: 11Thr/160Thr; C/G: 11Thr/160Ala; T/A: 11Met/Thr; T/G: 11Met/160Ala.

To confirm that SP-D 11Thr/160Thr (amino acid residues 11 and 160, both threonine) confer greater protection against TB infection as per the genetic analysis, as well as to further investigate the protective effects of four genetic variants of rSP-D in the progression of TB infection to TB disease, we constructed four genetic variants of SP-D, and then, respectively, measured their protective ability against *M. bovis* BCG *in vitro* (SP-D binds to the surface of pathogens as a first line of defense to prevent pathogens binding before TB infection). We analyzed the binding activities of the four genetic variants of rSP-D with *M. bovis* BCG and found that all four genetic variants of rSP-D bound to *M. bovis* BCG in a dose-dependent and specific manner that could be inhibited by 10 mM EDTA or 100 mM maltose, but not by 100 mM glucose (Figures 3A,B). At particular concentrations (1 µg/ml) of these four SP-D mutants, a change in the amino acid at residue 11 [from Met to Thr, i.e., rSP-D (92C/538A) and rSP-D (92C/538G)] led to higher binding activities with *M. bovis* BCG when compared with the other variants of rSP-D [rSP-D (92T/538A) and rSP-D (92T/538G)] (Figure 3B). Double reciprocal plot analyses revealed that the dissociation constant of rSP-D (92C/538A) binding to *M. bovis* was approximately sevenfold less than that for rSP-D (92T/538A) [4.5 × 10^9^ M for rSP-D (C/A), 3.1 × 10^8^ M for rSP-D (92T/538A)], indicating that the avidity of SP-D binding to *M. bovis* is affected by the change in amino acid at residue 11. By contrast, the change in amino acid residue 160 (from Thr to Ala) did not lead to a significant difference in binding ability to *M. bovis* BCG.

### Effects of the Four rSP-D Variants on Phagocytosis of MH-S Cells

**Figure 4:**
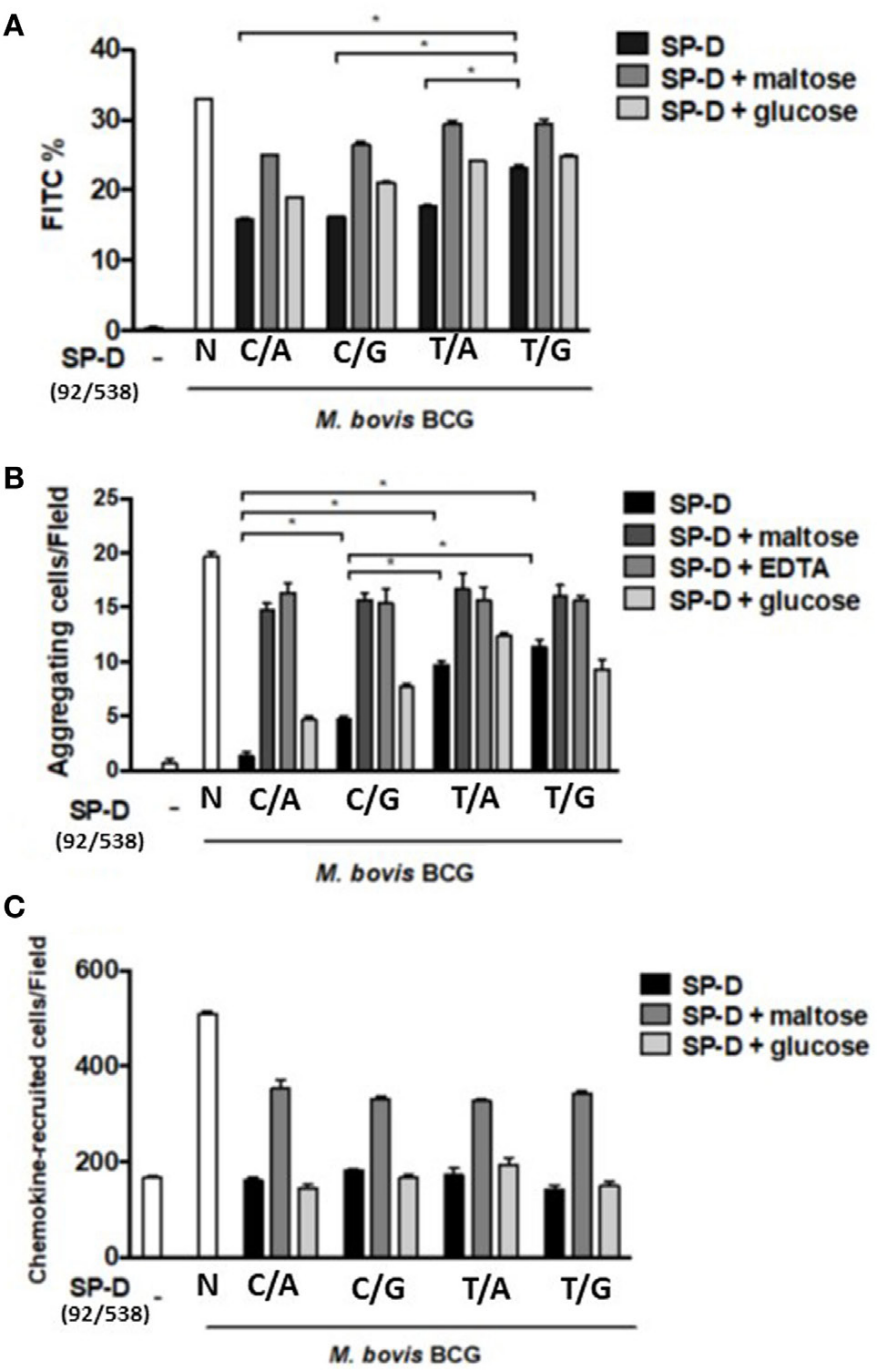
**(A)** Inhibition of alveolar macrophage phagocytosis of *Mycobacterium bovis* bacillus Calmette–Guérin (*M. bovis* BCG) by native and four genetic variants of recombinant SP-D (rSP-D) protein. Macrophages infected with FITC-labeled *M. bovis* BCG were analyzed using flow cytometry to assess the effects of the four rSP-D variants on the phagocytosis of MH-S cells. rSP-D with residue 11Thr [(92C/538A) and (92C/538G)] inhibited phagocytosis of *M. bovis* BCG by MH-S cells to a significantly greater extent than rSP-D with residue 11Met [(92T/538A) and (92T/538G)] (*P < 0.05). **(B)** Inhibition of agglutination of *M. bovis* BCG-infected MH-S cells by native and four genetic variants of rSP-D proteins. The method of assessment of agglutination of MH-S cells after mycobacterial infection is described in Section “Materials and Methods.” rSP-D with residue 11Thr [(92C/538A) and (92C/538G)] inhibited the aggregation of MH-S cells to a significantly greater extent than rSP-D with residue 11Met [(92T/538A) and (92T/538G)] (*P < 0.05). **(C)** Inhibition of cell migration of *M. bovis* BCG-infected MH-S cells by native and four genetic variants of rSP-D proteins. All four rSP-D variants inhibited cell migration of infected MH-S cells to nearby responsive cells in a concentration-dependent manner, but no significant difference was found between the four variants of rSP-D proteins. C/A: 11Thr/160Thr; C/G: 11Thr/160Ala; T/A: 11Met/Thr; T/G: 11Met/160Ala.

All rSP-D variants inhibited phagocytosis by alveolar macro_phages following *M. bovis* BCG infection, but with different potencies. As the results presented in Figure 4A show, the change in amino acid at residue 11 (from Met to Thr, i.e., 92T→92C) led to significant inhibition of phagocytosis. The SP-D variant 11M/160A (92T/538G) showed a weaker inhibition effect than the other variants (Figure 4A). The inhibitory effect of rSP-D could be attenuated by adding maltose as a competitor for bacterial binding.

### Determination of Aggregation and Chemotaxis of Infected MH-S Cells to Nearby Responsive Cells

Aggregation of infected MH-S cells, an event in which a collection of macrophages is formed during mycobacterial infection, was also evaluated. The rSP-D variant 11Thr/160Thr (92C/538A) inhibited the aggregation of infected MH-S cells more obviously than the other variants (Figure 4B). The protein with Thr at residue 11 [rSP-D (92C/538A) and (92C/538G)] showed a significantly stronger inhibitive effect than that with Met at residue 11 [rSP-D (92T/538A) and (92T/538G)]. The protein with Thr at residue 160 showed stronger inhibition than Ala 160 only when residue 11 was Thr and under the following conditions: 1 µg/ml SP-D or 1 µg/ml SP-D + 100 mM glucose.

We further analyzed the associations between aggregation of MH-S cells and various rSP-D mutants through a chemotaxis assay. However, the recruitment of nearby MH-S cells after mycobacterium infection did not differ between the four rSP-D variants (Figure 4C).

### The Associations Between the Four Variants of rSP-D and Viability of Infected MH-S Cells

**Figure 5:**
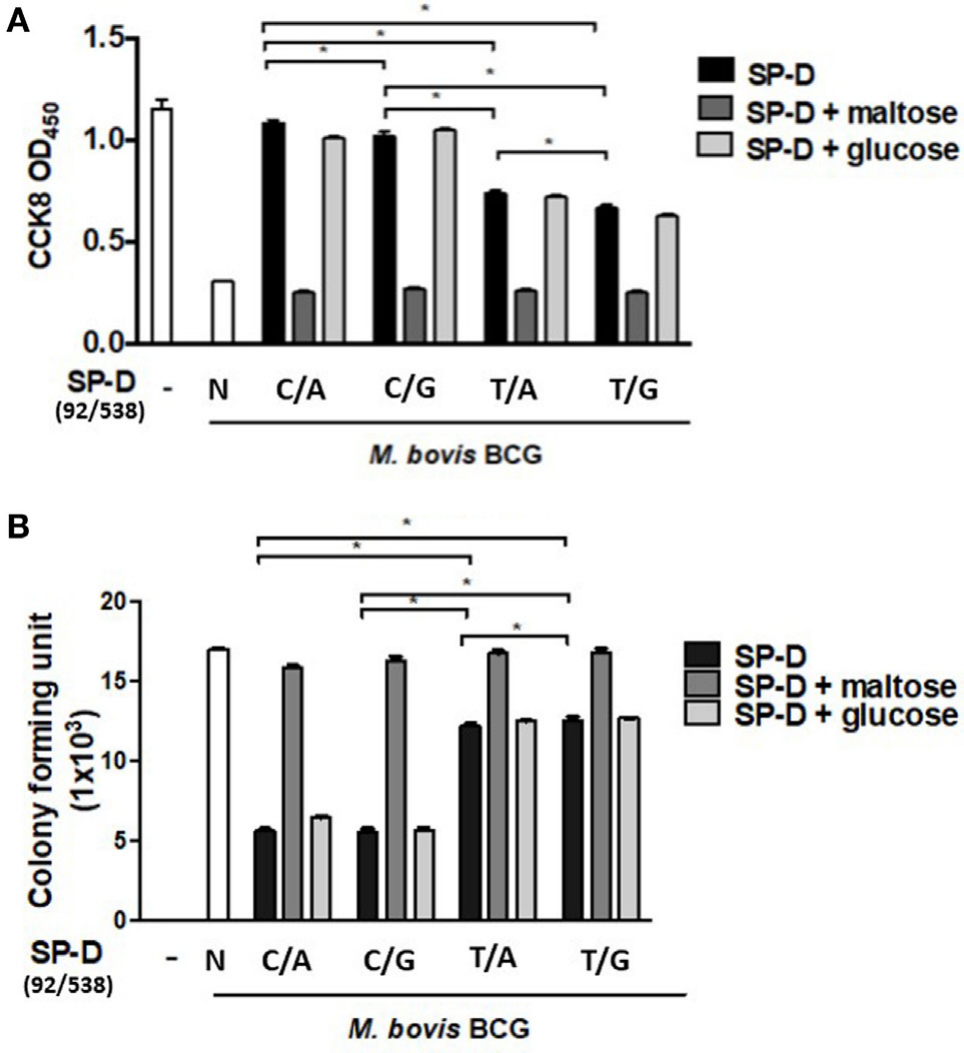
**(A)** Cell survival of *Mycobacterium bovis* bacillus Calmette–Guérin (*M. bovis* BCG)-infected MH-S cells by native and four genetic variant recombinant SP-D (rSP-D) proteins measured using a Cell Counting Kit-8 assay. rSP-D with residue 11Thr [(92C/538A) and (92C/538G)] exhibited significantly better survival of infected MH-S cells than rSP-D with residue 11Met [(92T/538A) and (92T/538G)] (*P < 0.05). **(B)** Intracellular variability of *M. bovis* BCG after phagocytosis by alveolar macrophages treated with native and four genetic variants of rSP-D proteins. rSP-D with residue 11Thr [(92C/538A) and (92C/538G)] inhibited *M. bovis* BCG intracellular growth significantly more than rSP-D with residue 11Met [(92T/538A) and (92T/538G)] (*P < 0.05). TT: 11T/160T; TA: 11T/160A; MT: 11M/160A; MA: 11M/160A.

When immune cells first encounter a pathogen in the host, macrophages are one of the major cell types to confront the bacterial invasion, and the response of the cells is crucial, as it controls the outcome of infection. To further elucidate the effect of rSP-D on MH-S cells infected by *M. bovis* BCG, a cell survival assay was performed using CCK-8. All variants of rSP-D improved the survival of infected MH-S cells in a concentration-dependent manner (Figure 5A). 1 µg/ml rSP-D with residue 11Thr [(92C/538A) and (92C/538G)] demonstrated the most significant protection of infected MH-S cells from cell death. In the rSP-D variants supplemented with 100 mM maltose, a protective effect was not found, and no change to cell survival was observed with treatment supplemented with 100 mM glucose.

### The Effects of rSP-D Variants on Intracellular *M. bovis* BCG Growth

The TB survival rate within macrophages is a hallmark of persistent (TB) pathogenesis. Aliquots of infected MH-S cell lysate were plated onto 7H11 agar to evaluate the effects of the four rSP-D variants on intracellular bacteria growth. Our results showed that all four variants of rSP-D inhibited intracellular *M. bovis* BCG growth (Figure 5B). The addition of 100 mM maltose reversed the inhibition, while no effect was observed with the addition of 100 mM glucose. Compared with residue 11Met [(92T/538A) and (92T/538G)], rSP-D variants with residue 11Thr [(92C/538A) and (92C/538G)] significantly inhibited the intracellular growth of *M. bovis* BCG (Figure 5B). There was no difference in growth whether residue 160 was bearing Ala or Thr.

## Discussion

In this study, we used *M. bovis* BCG, a good surrogate representing *MTB*, to study the role of SP-D polymorphism in mycobacterial infection and the connection with TB. Our results showed that *M. bovis* BCG interacted with the different rSP-D variants at various levels; these effects were abolished following the addition of maltose or EDTA, which causes a competitive effect with the lectin domain of SP-D or depletion of calcium. At first glance, it appeared that interaction of SP-D with *MTB* was dependent on its carbohydrate recognition domain (CRD). However, a closer look at the comparison of innate immunity against TB infection of genetic variants in the N-terminal and collagenous regions of rSP-D showed different degrees of activity. We found that SP-D residue 11 polymorphism, Thr (92C) and Met (92T), affected the binding activity of SP-D to *M. bovis* BCG, phagocytosis of infected MH-S cells, aggregation of infected MH-S cells, cell death of infected MH-S cells, and intracellular growth of *M. bovis* BCG. By contrast, the effects of SP-D 160 polymorphism, Ala (538G) and Thr (538A), were weaker than those of residue 11 polymorphism, and did not appear distinctly. From the results of SP-D variant functional and genetic analysis, residue 11 Met (92T) appeared likely to cause susceptibility to TB, which was validated in our TB-infected patients.

The SP-D gene contains a total of eight exons, seven of which are coding. Coding and non-coding SNPs (rs721917, rs6413520, rs2243639, rs3088308, rs1051246, rs1923537, rs2245121, rs911887, rs2255601, and rs7078012) are also found within the SP-D gene (31, 32); most of these have been found to be associated with a number of diseases (23, 33, 34). Among the known SP-D SNPs, two major polymorphisms, C92T (rs721917) and A538G (rs2243639), were chosen for analysis in this study. The rs721917 SFTPD SNP, known as C92T and SP-D A11, located on exon 1 and a short non-collagen-like N-terminal section, is associated with serum levels of SP-D (35), and explains 39% of the phenotypic variation (36), the Met11 (92T) allelic variant being associated at the highest level (37). Consistent with genetic determination of SP-D levels, the constitutive distribution of multimerized (high molecular weight, HMW) and non-multimerized (low molecular weight, LMW) SP-D in human body fluids is also genetically determined, with individuals homozygous for the Met11 allele having a relative predominance of HMW SP-D, and Thr11 (92C) allele homozygotes having more LMW SP-D (37). This change in the multimerization of SP-D may also affect its biological functions *in vitro* and *in vivo* (36). In fact, several studies have linked rs721917 with diseases (38), indicating involvement of SP-D level and/or size variation in disease pathogenesis. Study has shown that amino acid position 343 of SP-D has a critical role in the selective binding of glycolipids from *MTB* (39); however, no polymorphism exists in this genetic position.

Another coding single-nucleotide variation is located on exon 4, and the collagen-like region in the mature protein is rs2243639, known as A538G and SPD A160. As with other genetic alleles, the two-marker SP-D/SP-A haplotype, DA160_A/SP-A2 1A, is protective against the development of neonatal respiratory distress syndrome (40). SP-A2/SP-D haplotypes were also found to protect against bronchopulmonary dysplasia: 1A2-Ala160 (rs2243639) and 1A2-Thr11 (rs721917)-Ala160 (rs2243639) (41). It has been suggested that the protective effect of SP-D Ala160 in bronchopulmonary dysplasia is likely due to interactions with other gene(s). In our study of the interaction with *M. bovis* BCG, residue 160 Ala (92C/538G) showed a lesser binding activity than 160 Thr (92C/538A) when residue 11 was Thr, but not when it was Met. The SP-D variant 11M/160A (92T/538G) showed weaker inhibition of phagocytosis than the other variants at an SP-D concentration of 1 µg/ml. Our results implied that polymorphism A539G (160 residue Ala/Thr) has less impact than C92T (11 residue Thr/Met) and may exhibit an effect only under specific conditions or through interaction with other genetic factors. The protective effect of homozygous 538A (160 residue Thr) against TB was consistent with the results of functional studies mentioned above.

Surfactant proteins D binds to the bacterial surface and reduces the uptake of bacteria by alveolar macrophages (19). Also, SP-D polymorphisms have been demonstrated to be associated with the risk of TB (23, 42). A genetic study of a Mexican population identified that SP-D A11C (amino acid residue 11, risk allele threonine) was associated with subgroups of patients with TB by comparing a TB group and a tuberculin–skin test-positive group (symptom-free and normal chest radiographs). The association was not found when the TB group was compared with a control group (23). Our association study revealed that homozygous residue 11 Met was a risk genotype for TB, but this is not in agreement with the conclusions of the above study.

In this study, we demonstrated that the genetic variants of rSP-D in the N terminal and collagenous region confer different degrees of innate immunity against TB infection *in vitro*. Therefore, it is likely that SP-D polymorphisms C92T and A538G affect susceptibility to TB, which is in agreement with our findings in an association study of clinical TB patients. There were some limitations to our study. First, we were not able to differentiate whether or not these genetic variants of rSP-D undergo multimerization (HMW vs LMW) under our experimental conditions. HMW SP-D multimers are only partly dependent on disulfide crosslinking of the N terminal region, and some SP-D subunits are non-covalently associated depending on the protein concentration in the solution (36). Several studies have shown some discrepancies in binding characteristics and activities between HMW and LMW SP-D (36, 37, 43). Second, we were not able to demonstrate *in vivo* effects of the genetic variants of rSP-D in a TB-infected animal model due to a small yield of recombinant full-length human SP-D from HEK 293 cells. Third, although high antigenic and structural similarities exist between *MTB* and *M. bovis* BCG, use of *M. bovis* might not wholly represent the infectious nature of *MTB* in hosts. *MTB* could not be utilized in this study due to a lack of availability of the necessary safety equipment in our laboratory.

However, to evaluate the influences of SP-D polymorphisms *in vivo* and in lung diseases, wild-type and transgenic *Sftpd*^−/−^ mice expressing either the human SP-D Met11 or Thr11 allelic variants were generated (44–46). In an acute model of allergic asthma, transgenic mice were sensitized and challenged with ovalbumin, and the results showed that murine expression of human polymorphic SP-D variants in the N-terminal region does not significantly influence the severity of allergic airway inflammation (46). Other studies also showed that low transgene expression levels and differences in the distribution of human rSP-D in the lung made comparison of phenotypes between wild-type and transgenic mice difficult (44, 45). In fact, a 60-kDa recombinant trimeric fragment of SP-D, without the N-terminus but maintaining part of the collagen region, appeared to have comparable effects to native SP-D in *in vivo* and *in vitro* studies, which implies that the homeostatic and anti-microbial effects of SP-D are primarily mediated by its CRD region (47, 48).

In conclusion, our results demonstrated the role of SP-D in innate immunity against TB infection. Although this mostly depends on the binding of CRD with *MTB*, this study elucidates that it is also influenced by the genetic variants in the N-terminal and collagenous domains of SP-D. The two major SP-D polymorphisms C92T and A538G, and especially C92T, appeared to affect susceptibility to TB. Our association study of the susceptibility to TB infection in patients also supported this hypothesis.

## Ethics Statement

The study protocol conformed to the ethical guidelines of the 1975 Declaration of Helsinki and was approved by the Ethics Committees of Taoyuan General Hospital and National Cheng Kung University Hospital.

## Author Contributions

M-HH, C-YO, W-YH, J-YW, and LW contributed to the conception and design of the study; M-HH and W-YH performed experiments; C-YO and H-FK analyzed the data; C-YO and S-WL collected TB patients and performed genetic analysis; LW and J-YW wrote the first draft of the manuscript; and all authors contributed to manuscript revision, and read and approved the submitted version.

## Conflict of Interest Statement

The authors declare that the research was conducted in the absence of any commercial or financial relationships that could be construed as a potential conflict of interest.

## Abbreviations

CRD, carbohydrate recognition domain; HMW, high molecular weight; LMW, low molecular weight; LAM, lipoarabinomannan; *MTB, Mycobacterium tuberculosis*; MOI, multiplicity of infection; rSP-D, recombinant SP-D; SNPs, single-nucleotide polymorphisms; SP-D, surfactant proteins D; TB, tuberculosis; *M. bovis* BCG, *Mycobacterium bovis* bacillus Calmette–Guérin.
